# Exploring gender impact on collaborative care planning: insights from a community mental health service study in Italy

**DOI:** 10.1186/s12888-023-05307-5

**Published:** 2023-11-13

**Authors:** Alessandra Martinelli, Chiara Bonetto, Tecla Pozzan, Elena Procura, Doriana Cristofalo, Mirella Ruggeri, Helen Killaspy

**Affiliations:** 1 IRCCS Istituto Centro San Giovanni Di Dio Fatebenefratelli, Brescia, Italy; 2 Department of Neurosciences, Biomedicine and Movement Sciences, University of Verona, Verona, Italy; 3 Azienda Ospedaliera Universitaria Integrata Verona, Verona, Italy; 4 Mental Health Center, Isola Della Scala, Ospedale Di Bussolengo, Verona, Italy; 5 Division of Psychiatry, University College London, London, UK

**Keywords:** Personal recovery, Goals, Gender, Community case study, Recovery star

## Abstract

**Introduction:**

Personal recovery is associated with socio-demographic and clinical factors, and gender seems to influence the recovery process. This study aimed to investigate: i) differences in the recovery goals of men and women users of a community mental health service in Italy; ii) any differences by gender in recovery over six months using the Mental Health Recovery Star (MHRS).

**Methods:**

Service users and staff completed the MHRS together at recruitment and six months later to agree the recovery goals they wished to focus on. Socio-demographic and clinical characteristics and ratings of symptoms (BPRS), needs (CAN), functioning (FPS), and functional autonomy (MPR) were collected at recruitment and six months follow-up. Comparisons between men and women were made using t-tests.

**Results:**

Ten women and 15 men completed the MHRS with 19 mental health professionals. Other than gender, men and women had similar socio-demographic, and clinical characteristics at recruitment. Women tended to choose recovery goals that focused on relationships whereas men tended to focus on work related goals. At follow-up, both men and women showed improvement in their recovery (MHRS) and women were less likely to focus on relationship related goals, perhaps because some had found romantic partners. There were also gains for both men and women in engagement with work related activities. Ratings of functional autonomy (MPR) improved for both men and women, and men also showed improvement in symptoms (BPRS) and functioning (FPS).

**Conclusions:**

Our findings suggest that collaborative care planning tools such as the MHRS can assist in identifying individualized recovery goals for men and women with severe mental health problems as part of their rehabilitation.

## Introduction

Personal recovery is a positive approach to mental health that empowers people with longer term and more severe mental disorders to live satisfying and rewarding lives, despite ongoing psychiatric symptoms and associated impairments in functioning [1–7]. Key principles are the promotion of hope, self-determination, self-efficacy [3] and empowerment through a collaborative process that includes shared-decision making [4–6], evidence-based [7, 8] and person-centered [4] practice. There is a focus on identifying and building on people’s strengths, preferences, and aspirations through an equal partnership between the person with mental health problems and the mental health professionals supporting them [3–6, 9]. This process may not be straight forward and is often characterized by new discoveries as well as setbacks [10, 11].

The personal recovery approach promotes people’s human rights and has been found to be associated with improvements in symptoms, functioning, social and life skills, quality of life and satisfaction with care, as well as reductions in care needs [3, 12–15]. As a result, recovery-based practice is recognized [10] as a key component of effective rehabilitation [16, 17].

An important aspect to be considered in personal recovery is gender and the socio-cultural constructs encompassing behaviours, attitudes, rules, and norms that are associated with being men or women. Gender differs from sex, which refers to a set of biological and physiological characteristics [18]. Both are relevant to the impact of mental health problems on an individual and the way in which mental health services may need to respond to them.

Gender norms and biases are culture-based assumptions about the life experiences, expected behaviors, interests, and choices of men and women [19, 20]. Studies in other medical fields have demonstrated gender bias [21] in the patient-professional encounter and the choice of treatments offered to men and women [22–26]. This may be due to assumptions about how men and women express and cope with symptoms. For example, classically, boys are expected to hide distress, whereas it is more permissible for girls to talk about their feelings [27]. Gender awareness is therefore an important area of competence for health professionals [19] in order to provide appropriate care that meets the needs of all patients [22].

Some studies have investigated whether personal recovery outcomes are influenced by gender. Results are inconsistent, with some reporting that better recovery outcomes were achieved by women, while others report that recovery-oriented interventions were beneficial regardless of gender [20, 28–31]. Some studies have investigated whether there may be any gender bias and/or influence in recovery orientated practice [32–37]. However, to date, only one pilot study has been published describing the provision of gender-sensitive and recovery-oriented interventions for women [38].

This small, exploratory study aimed to investigate, for the first time in Italy, gender bias in recovery orientated practice by looking into: i) differences in the recovery goals identified by men and women of a community mental health service (CMHS) in Verona Italy, that uses an established recovery orientated care planning tool to facilitate shared-decision making between the user and key professional/case manager, the Mental Health Recovery Star (MHRS) [39–44], ii) differences between men and women in regard to the progress they make in their recovery over six months.

## Methods

### Study design

Written informed consent for participation in the study was obtained from all service users and mental health professionals.

This small, prospective cohort study was conducted from May 2017 to October 2018 in the CMHS based in the South of Verona. Data were gathered at participant recruitment and six-month follow-up. The follow-up was scheduled at 6 months to coincide with the evaluation time typically provided in Italian rehabilitation settings, such as day centers and supported housing. It was envisaged that relatively small numbers of participants would be recruited, given the sampling pool size, and thus the study was designed to be exploratory.

### Participants

Mental health professionals meeting the following criteria were eligible to participate in the study: 1) work at the South Verona CMHS; 2) have been trained in the use of the MHRS; 3) willing to participate; 4) able to recruit at least one service user for whom they were the key professional or case manager (this involves drawing up care plans with the service users, co-orientating care as needed, and acting as the main contact for family members and other carers).

**Fig. 1:**
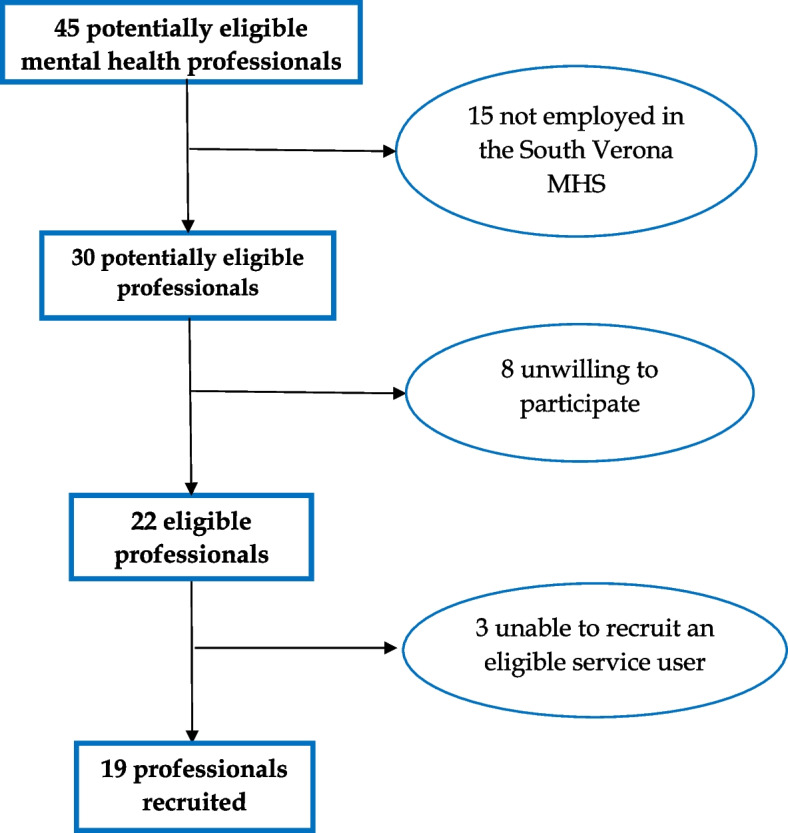
Flowchart showing mental health professional recruitment into study

Forty-five professionals participated in a three-day training course in the use of the MHRS between May 2017 and October 2017. Nineteen of the 45 were recruited to the study (15 did not work in an eligible CMHS; 8 declined to participate; 3 were unable to recruit at least one service user into the study) (Fig. 1). Most of the professional participants were female (79.0%) and over a third (37%) were trainee psychiatrists. Most (56%) worked in community based multidisciplinary teams and the mean length of time they had worked there was 137 (SD 122.2) months (Table 1). Mental health professional participants received monthly supervision from a certified MHRS trainer and staff experienced in recovery-oriented rehabilitation. This ensured consistency between practitioners in the use of the MHRS and encouraged recovery-oriented practices, such as motivational interviewing, negotiation skills, person-centred care planning, and agreeing specific recovery goals with clients [16].

**Table 1:** Mental health professionals’ demographic and work characteristics

Mental health professionals	19
*Gender, Male*	4 (21%)
*Discipline*	
Trainee psychiatrist	7 (37%)
Support worker	3 (16%)
Nurse	4 (21%)
Psychologist	2 (10%)
Vocational worker	2 (10%)
Non-professional support staff (in Italian ‘OSS’ – these staff have a qualification in health and social care)	1 (5%)
*Mean (SD) months working in mental health* *Work setting*	137 (122.2)
Community multidisciplinary team	14 (56%)
Supported housings	4 (16%)
Day Centre	6 (24%)
Other	1 (4%)

Service users were recruited to the study according to the following eligibility criteria: 1) have a key professional/case manager trained in the use of the MHRS; 2) living in the community within the catchment area of the South Verona Mental Health Service; 3) aged between 18 and 65 years; 4) willing to complete a range of assessments at two time-points. Exclusion criteria were: 1) diagnosis of moderate or severe intellectual disability [54]; 2) being an inpatient in the psychiatric ward or at risk of being admitted to the ward due to the severity of their symptoms during the study recruitment period (May 2017 to May 2018). Eligible service users were identified for potential participation in the study by a key professional or case manager, who explained the purpose of the study and gained written informed consent from them. A total of 25 service users were recruited.

### Assessments

Socio-demographic, service use and other clinical data on service user participants were obtained from the Verona Mental Health Department database and South-Verona Psychiatric Case Register-PCR [45]. Service users' gender was recorded according to self-identification as a man or woman from the case records. At baseline and follow-up, the service users’ key professional completed the following standardised assessments:

- *Personal and social functioning* was assessed using the Personal and Social Functioning Scale (FPS) [46, 47] which examines four main areas: socially useful activities (including work and study), personal and social relationships (including relationships with family), self-care and hygiene, disturbing and aggressive behaviour. The total score ranges from 1 to 100 with higher numbers indicating better functioning.
- *Psychopathology and social functioning* were assessed using the Health of the Nation Outcome Scale (HoNOS) [48, 49] which consists of 12 items covering 4 areas (behaviours that impact negatively on the person and/or others; problems with managing day to day activities; symptoms of mental ill-health that distress or limit the person; social, housing and/or occupational problems that limit autonomy). Scores are given on 5-point Likert scale (0 = no problem; 4 = very severe problem). The total score is the sum for all items, thus ranging from 0 to 48, with a higher value reflecting greater severity of psychopathology or lower functioning.
- *Functional autonomy* was assessed using the Monitoring of the Path of Rehabilitation (MPR) [50]. This tool provides an assessment of the person's ability to perform independently various activities of daily living [51] including self-care, housework, shopping, cooking, using public transport, accessing community activities (social, leisure), engaging in occupational activities, and managing physical and mental health. The total mean score ranges from 0 to 12, with higher scores indicating greater functional autonomy.
- *Needs for care* were assessed using the Italian version of the Camberwell Assessment of Need (CAN) staff version [52, 53], which comprises 22 items grouped into four domains (health, basic, service, and functioning). Each item was assessed as 0 = no problem, 1 = no⁄moderate problem (met need), 2 = current severe problem (unmet need). The number of needs (scores of 1 or 2) and unmet needs (scores of 2) is aggregated over the 22 items and the ratio of met:unmet needs calculated.
- *Personal recovery process and recovery goals* were assessed using the Italian version of the MHRS [54, 55]. MHRS was developed via participatory action research involving researchers, service users, mental health professionals, and informal caregivers. Its purpose is to facilitate and monitor the process of personal recovery. The MHRS comprises a 10-point star-shaped visual schema where each point corresponds to a life dimension, with these further grouped into four dimensions: physical and mental health (managing mental health, self-care, addictive behaviour); activities and functioning (living skills, work, responsibilities); self-image (identity and self-esteem, trust and hope); networks (social networks, relationships). Through discussion with their key professional, service users are supported to rate their progress on each domain on a ten-point ‘Scale of Change’, which describes five steps in the recovery process, each sub-divided into two phases as follows:
- Stuck (phases 1–2): feeling unable to cope with the problem or not being able to accept help for it.
- Accepting help (phases 3–4): the desire to get away from the problem and the hope that someone/something can intervene to assist.
- Believing (phases 5–6): starting to believe in the possibility of change, starting to do things to achieve personal goals and accepting help from others.
- Learning (phases 7–8): actively trying things out and learning through trial and error with support.
- Self-reliance (phases 9–10): being able to achieve and manage the desired goal/s without support.

After ratings are completed, the key professional and client discuss and agree together specific recovery goals and, through shared decision making, a care plan to support them to achieve them. A maximum of three goals are worked on at a time [32].

### Statistical analysis

Descriptive data were presented as frequencies, means and standard deviations. The Kolmogorov–Smirnov test was applied for all the continuous variables and their distributions were found to be Normal, with the exception of Total unmet needs. Thus, parametric tests (t test for independent and paired groups) were used for all continuous variables. The Total unmet needs comparisons were also performed with non-parametric tests (Mann–Whitney and Wilcoxon, respectively), but no differences were found with respect to the corresponding parametric tests, which are shown in tables. Comparisons between men and women service users at recruitment were made using t-tests for independent samples. Changes between scores on the standardised assessment tools between recruitment and follow-up were investigated using the t-test for repeated measurements (paired samples correlations). All tests were bilateral with a significance level set at 0.05. Statistical analyses were performed using the SPSS 22.0 program.

### Power of the study

Although this was an exploratory study, we conducted a sample size estimate using Stata 13.0 software to detect a statistically significant improvement in MHRS Scale of Change scores between recruitment and six-month follow-up. A prospective study that used the MHRS in 15 London mental health services [56] reported a mean improvement of 0.26 in the MHRS Scale of Change score over 3.5 months (increasing from 5.22 to 5.48). Assuming a difference in standard deviation less than or equal to 0.40, a power of 80% and an alpha level of 0.05, a sample size of 21 was required to detect a similar improvement in MHRS scores. Assuming a maximum 20% drop-out, we aimed to recruit 25 participants.

## Results

### Service user participants’ socio-demographic and clinical characteristics at recruitment

As shown in Table 2, the sample comprised 25 service users. Their mean age was 41.1 (SD = 9.9) years and most were single (76.0%), unemployed (40.0%), and living with family (52.0%). Most had been diagnosed with a psychotic illness (68.0%) and had a mean length of contact with mental health services of 16.0 (SD = 8.4) years. Over one quarter (28.0%) had been admitted to an acute psychiatric ward in the previous year. One-third (32.0%) had some form of physical health comorbidity and almost half (48.0%) were considered to have a substance misuse problem. Ten (40%) service users identified as women and 15 (60%) as men.

**Table 2:** Service users’ sociodemographic and clinical characteristics, functioning, psychopathology, functional autonomy and needs at recruitment

	**Women** **(*N* = 10)**	**Men** **(*N* = 15)**	**Total** **(*N* = 25)**	***P*-value**
Sociodemographic and clinical characteristics				
*Mean (SD) age in years*	41.8 (12.1)	40.7 (8.6)	41.1 (9.9)	0.786
*Marital status*				-
Single	7 (70.0%)	12 (80.0%)	19 (76.0%)	
Partnered	3 (30.0%)	3 (20.0%)	6 (24.0%)	
*Educational achievement*				-
Lower education (primary/middle school only) Higher education (high school/further education)	7 (70.0%) 3 (30.0%)	6 (40.0%) 9 (60.0%)	13 (52.0%) 12 (48.0%)	
*Work*				-
Employed	2 (20.0%)	2 (13.3%)	4 (16.0%)	
Unemployed	1 (10.0%)	9 (60.0%)	10 (40.0%)	
Supported employment	2 (20.0%)	1 (6.7%)	3 (12.0%)	
Student	1 (10.0%)	1 (6.7%)	2 (8.0%)	
Homemaker	1 (10.0%)	0 (0.0%)	1 (4.0%)	
Retired	3 (30.0%)	2 (13.3%)	5 (20.0%)	
*Housing*				-
Alone	1 (10.0%)	0 (0.0%)	1 (4.0%)	
With family/partner	5 (50.0%)	8 (53.3%)	13 (52.0%)	
Supported housings	11 (40.0%)	7 (46.7%)	11 (44.0%)	
*Primary clinical diagnosis*				-
Schizophrenia	2 (20.0%)	6 (40.0%)	8 (32.0%)	
Other psychosis (schizoaffective disorder, delusional disorder)	4 (40.0%)	5 (33.3%)	9 (36.0%)	
Affective disorders (bipolar affective disorder, depression)	2 (20.0%)	0 (0.0%)	2 (8.0%)	
Other diagnoses (e.g. personality disorders)	2 (20.0%)	4 (26.6%)	6 (24.0%)	
*Mean (SD) years of contact with community mental health service*	16.3 (8.7)	15.9 (8.4)	16.0 (8.4)	0.902
*Psychiatric admission in the last year*				-
No admission	7 (70.0%)	11 (73.3%)	18 (72.0%)	
1 voluntary admission	3 (30.0%)	3 (20.0%)	6 (24.0%)	
1 involuntary admission	0 (0.0%)	1 (6.7%)	1 (4.0%)	
*Mean (SD) length in months of admissions in the last year*	55.7 (28.2)	23 (3.5)	37.0 (24.0)	0.064
*Physical health comorbidity (e.g. dyslipidemia, hypothyroidism)*	5 (50.0%)	3 (20.0%)	8 (32.0%)	-
*Substance misuse or gambling problem*	3 (30.0%)	9 (60.0%)	12 (48.0%)	-
*Rating scale assessments*				
Mean (SD) functioning (FPS) [0, very severe dysfunction; 90 very good functioning]	62.1 (20.3)	46.2 (17.1)	53 (19.7)	**0.046**
Mean (SD) psychopathology and social functioning (HoNOS) [0 = no problems at all; 48 = severe problems in all areas]	11.4 (6.5)	13.4 (4.8)	12.6 (5.5)	0.386
Mean (SD) functional autonomy (MPR) [0 = non-autonomous; 12 = fully autonomous]	9.2 (1.3)	8.3 (1.5)	8.7 (1.5)	0.108
Total, met and unmet needs (CAN) [0 = no problem, 1 = no ⁄ moderate problem (met need), 2 = current severe problem (unmet need)]				
Ratio met/unmet ≥ 1 indicates more needs are met than unmet				
Total mean (SD) needs	7.9 (5.0)	11.4 (3.9)	10.8 (4.5)	0.095
Total (SD) met needs	5.6 (3.7)	8.6 (3.7)	7.7 (3.7)	
Total (SD) unmet needs	2.3 (2.9)	2.9 (2.7)	3.0 (0.54)	0.184
Ratio met/unmet needs	2.4	3.0	2.6	0.347 -

^*^p-value < 0.05—Bold values denote statistical significance at the *p* < 0.05 level

Fewer women (30%) than men (60%) had completed higher education. Fewer women (20%) than men (40%) had a diagnosis of schizophrenia and a greater proportion of men (60%) than women (30%) had a substance misuse problem. Men and women had a similar length of contact with mental health services and a similar proportion had a recent admission (Table 2).

### Functioning, psychopathology, functional autonomy and care needs at recruitment

At recruitment, ratings of service users’ functioning and psychopathology suggested a group with severe mental health problems as reflected in the total mean scores on the FPS [53 (SD = 19.7)], HoNOS [12.6 (SD = 5.5)] and MPR [8.7 (SD = 1.5)]. Similarly, on average, they had a high level of needs as assessed by the CAN [mean 10.8 (SD = 4.5)] though most needs were met (ratio met:unmet needs: 2.6) (Table 2). There were no statistically significant differences in these ratings between men and women service users.

### Differences in men and women’s recovery

As shown in Table 3, at recruitment, on average, participants were rated at the ‘believing phase’ in their recovery [MHRS, mean 6.1 (SD = 1.5)]. In terms of individual MHRS dimensions, the lowest score for the Scale of Change was found in the domain of ‘networks’ [mean 5.4 (SD = 1.9)] while the highest score was found in the ‘physical and mental health’ domain [mean 6.6 (SD = 1.7)].

**Table 3:** Service users’ mean (SD) MHRS scale of change total, domain and sub-domain scores at recruitment [1–2 Stuck, 3–4 Accepting help, 5–6 Believing, 7–8 Learning, 9–10 Self-reliance]

	**Women** **(*N* = 10)**	**Men** **(*N* = 15)**	**Total** **(*N* = 25)**	***P*-value**
*Total mean (SD) MHRS scale of change*	7.0 (1.5) *n* = 9	5.6 (1.2)	6.1 (1.5) *n* = 24	**0.018**
**Physical and mental health**	7.7 (1.3)	5.8 (1.5)	6.6 (1.7)	**0.003**
Managing mental health	6.6 (1.6)	5.5 (1.7)	5.8 (1.8)	0.115
Self-care	8.0 (1.8)	6.1 (2.4)	6.7 (2.3)	**0.040**
Addictive behavior	8.5 (2.3)	5.9 (3.5)	7.0 (3.4)	0.050
**Activities and functioning**	7.6 (1.9) *n* = 9	5.7 (1.2)	6.4 (1.7) *n* = 24	**0.022**
Living skills	7.0 (2.2)	5.3 (1.5)	6.0 (2.0)	**0.029**
Work	6.8 (3.2) *n* = 9	4.5 (1.3)	5.5 (2.4) *n* = 24	**0.021**
Responsibilities	9.1 (2.0)	7.3 (2.5)	8.1 (2.3)	0.050
**Self -image**	6.6 (1.9)	5.6 (1.5)	6.0 (1.7)	0.175
Identity and self-esteem	6.6 (1.8)	5.5 (1.7)	6.0 (1.8)	0.126
Trust and hope	6.6 (2.0)	5.7 (1.7)	6.9 (1.9)	0.253
**Networks**	5.9 (1.9)	5.0 (1.9)	5.4 (1.9)	0.264
Social networks	6.5 (2.7)	4.5 (1.5)	5.5 (2.2)	**0.029**
Relationships	5.3 (2.4)	5.5 (2.8)	5.5 (2.6)	0.878

^*^p-value < 0.05 Bold values denote statistical significance at the *p* < 0.05 level

Overall, women scored higher on the MHRS Scale of Change than men at recruitment i.e. they were further on in the process of recovery (women were in the ‘Learning’ phase and men were in the ‘Believing’ phase). They also had higher scores than men on the Scale of Change in the dimensions of ‘physical and mental health’ and its sub-domain ‘self-care’, in the domain of ‘activities and functioning’ and its sub-domains ‘living skills’ and ‘work’, and in the sub-domain ‘social networks’ (Table 3).

As shown in Table 4, at recruitment, the collaborative discussions between service users and their key professional/case manager most commonly led to an agreement to focus on the achievement of personal goals in the dimension’activities and functioning’ (18, 72.0%). The least common domain chosen to focus on was ‘self-image’ (3,12%). However, there were differences between men and women in the areas chosen. Women tended to focus their intervention plans on the domain of 'networks' (8, 80.0%), and particularly when they focused on the subdomain of 'relationships' (5, 50.0%), they all reported wanting to develop a romantic relationship with a specific person. Men tended to focus on the dimension ‘activities and functioning’ (13, 86.7%), particularly in the sub-domain of work (8, 53.3%) (Table 4).

**Table 4:** Choice of MHRS domains and sub-domains for focussed care planning by gender at recruitment and follow-up

	**Recruitment**			**6-month follow-up**		
	**Women** **(*N* = 10)**	**Men** **(*N* = 15)**	**Total** **(*N* = 25)**	**Women** **(*N* = 10)**	**Men** **(*N* = 15)**	**Total** **(*N* = 25)**
***Area/s of intervention***						
**Physical and mental health**	3 (30.0%)	8 (53.3%)	11 (44.0%)	2 (20.0%)	12 (85.7%)	14 (70.0%)
Managing mental health	2 (20.0%)	3 (20.0%)	5 (20.0%)	1 (16.7%)	4 (28.6%)	5 (25.0%)
Self-care	1 (10.0%)	4 (26.7%)	5 (20.0%)	1 (16.7%)	4 (28.6%)	5 (25.0%)
Addictive behavior	0 (0.0%)	1 (6.7%)	1 (4.0%)	0 (0.0%)	4 (28.6%)	4 (20.0%)
**Activities and functioning**	5 (50.0%)	13 (86.7%)	18 (72.0%)	4 (40.0%)	12 (85.7%)	16 (80.0%)
Living skills	1 (10.0%)	3 (20.0%)	4 (16.0%)	2 (33.3%)	3 (21.4%)	5 (25.0%)
Work	4 (40.0%)	8 (53.3%)	12 (48.0%)	2 (33.3%)	7 (50.0%)	9 (45.0%)
Responsibilities	0 (0.0%)	2 (13.3%)	2 (8.0%)	0 (0.0%)	2 (14.3%)	2 (10.0%)
**Self-image**	1 (10.0%)	2 (13.3%)	3 (12.0%)	2 (33.3%)	1 (7.1%)	3 (15.0%)
Identity and self-esteem	1 (10.0%)	1 (6.7%)	2 (8.0%)	2 (33.3%)	1 (7.1%)	3 (15.0%)
Trust and hope	0 (0.0%)	1 (6.7%)	1 (4.0%)	0 (0.0%)	0 (0.0%)	0 (0.0%)
**Networks**	8 (80.0%)	4 (26.7%)	12 (48.0%)	3 (30.0%)	7 (50.0%)	10 (50.0%)
Social networks	3 (30.0%)	2 (13.3%)	5 (20.0%)	1 (16.7%)	4 (28.6%)	5 (25.0%)
Relationships	5 (50.0%)	2 (13.3%)	7 (28.0%)	2 (33.3%)	3 (21.4%)	5 (25.0%)

### Change in socio-demographic, clinical characteristics, functioning, psychopathology and functional autonomy from recruitment to 6 months follow up

As shown in Table 5, between recruitment and 6-month follow up, two women and one man found romantic partners, one woman and three men started to engage, and one man gained employment. Both men and women showed statistically significant improvements in ratings of their functional autonomy (MPR) and men also showed improvements in their ratings of clinical status (HoNOS) and functioning (FPS). The total mean number of needs of all participants reduced (women from 7.1 [SD 4.9] to 5.9 [SD 3.7]; men from 11.4 [SD 3.9] to 10.3 [SD = 4.4]) but these improvements were not statistically significant. However, there was an increase in the ratio of met:unmet needs for both women (2.4 to 7.4) and men (2.9 to 4.5) (Table 5).

**Table 5:** Change in service users’ sociodemographic and clinical characteristics, process of recovery (MHRS), functioning (FPS), psychopathology (HoNOS) and functional autonomy (MPR) by gender from recruitment to 6 month follow up

	**Recruitment** **Women** **(*N* = 10)**	**6 month follow up Women (*N* = 10)**	***p*-value** **paired t-test**	**Recruitment** **Men** **(*N* = 15)**	**6 month follow up Men** **(*N* = 15)**	***p*-value paired t-test**
***Sociodemographic and clinical characteristics***						
***Marital status***						
**Single**	7 (70.0%)	5 (50.0%)	-	12 (80.0%)	11 (73.3%)	
**Partnered**	3 (30.0%)	5 (50.0%)		3 (20.0%)	4 (26.7%)	-
***Education***						
**Lower education (primary/middle school)**	7 (70.0%)		-	6 (40.0%)		-
**Higher education (high school/ further education)**	3 (30.0%)	7 (70.0%) 3 (30.0%)		9 (60.0%)	6 (40.0%) 9 (60.0%)	
***Work***						
**Employed**	2 (20.0%)	2 (20.0%)	-	2 (13.3%)	3 (20.0%)	-
**Unemployed**	1 (10.0%)	0 (0.0%)		9 (60.0%)	5 (33.3%)	
**Supported employment**	2 (20.0%)	3 (30.0%)		1 (6.7%)	4 (26.7%)	
**Student**	1 (10.0%)	1 (10.0%)		1 (6.7%)	1 (6.7%)	
**Homemaker**	1 (10.0%)	1 (10.0%)		0 (0.0%)	0 (0.0%)	
**Retired**	3 (30.0%)	3 (30.0%)		2 (13.3%)	2 (13.3%)	
***Housing***	1 (10.0%)	2 (20.0%)	-	0 (0.0%)	0 (0.0%)	
**Alone**	5 (50.0%)	5 (50.0%)		8 (53.3%)	7 (46.7%)	
**With family/partner** **Supported accommodation**	4 (40.0%)	3 (30.0%)		7 (46.7%)	8 (53.3%)	
***Substance misuse or gambling problem***	3 (30.0%)	3 (30.0%)	-	9 (60.0%)	9 (60%)	-
***Assessments***						
**Mean (SD) functioning (FPS)** [0, very severe dysfunction; 90 very good functioning]	62.1 (20.3)	66.0 (15.9)	0.311	46.2 (17.1)	51.3 (16.3)	**0.013**
**Mean (SD) psychopathology and social functioning (HoNOS)** [0 = no problems at all; 48 = severe problems in all areas]	11.4 (6.5)	8.1 (4.1)	0.086	13.4 (4.8)	10.7 (4.7)	**0.003**
**Mean (SD) functional autonomy (MPR)** [0 = non-autonomous; 12 = fully autonomous]	9.2 (1.3)	9.9 (1.4)	**0.013**	8.3 (1.5)	9.0 (1.5)	**0.042**
**Total, met and unmet needs (CAN)** [0 = no problem, 1 = no ⁄ moderate problem (met need), 2 = current severe problem (unmet need)]						
Ratio met/unmet ≥ 1 indicates more needs are met than unmet	7.1 (4.6) *n* = 9	5.9 (3.7)	0.315	11.4 (3.9)	10.3 (4.4)	0.164
**Total mean (SD) needs**	5.0 (3.4) *n* = 9	5.2 (3.4)	0.760	8.5 (3.7)	8.5 (4.2)	0.939
**Total (SD) met needs**	2.1 (3.0) *n* = 9	0.7 (1.3)	0.182	2.9 (2.7)	1.9 (1.8)	0.087
**Total (SD) unmet needs** **Ratio met/unmet needs**	2.4	7.4	-	2.9	4.5	-
**Mean (SD) Process of Change (MHRS) score** [1–2 Stuck, 3–4 Accepting help, 5–6 Believing, 7–8 Learning, 9–10 Self-reliance]	7.2 (1.5)	7.6 (1.6) *n* = 8	0.245	5.6 (1.2)	6.1 (1.1)	**0.006**
**Mean (SD) MHRS physical and mental health**	7.7 (1.4)	7.9 (1.4)	0.776	5.8 (1.5)	6.4 (1.2)	**0.018**
Managing mental health	6.6 (1.7)	7.3 (1.9) *n* = 9	**0.023**	5.5 (1.7)	6.2 (1.4)	0.052
Self-care	7.8 (1.7)	8.0 (1.7) *n* = 9	0.347	6.1 (2.4)	6.8 (2.2)	0.102
Addictive behavior	8.9 (2.1)	8.2 (3.0) *n* = 9	0.493	4.5 (1.5)	4.9 (1.8)	0.068
**Mean (SD) MHRS activities and functioning**	7.9 (1.7)	8.1 (1.6)	**0.049**	5.7 (6.3)	6.3 (1.1)	**0.009**
Living skills	7.2 (2.2)	7.6 (1.8) *n* = 9	0.195	5.3 (1.5)	5.9 (1.6)	**0.036**
Work	7.4 (2.9)	7.8 (2.9) *n* = 8	0.197	6.1 (2.4)	6.8 (2.2)	**0.023**
Responsibilities	9.3 (1.0)	9.2 (1.0) *n* = 9	0.347	4.5 (1.3)	5.1 (1.4)	**0.015**
**Mean (SD) MHRS self-image**	6.7 (2.0)	7.3 (1.9)	0.102	5.6 (1.5)	6.1 (1.6)	0.100
Identity and self-esteem	6.7 (1.9)	7.0 (1.9) *n* = 9	0.347	5.5 (1.7)	6.1 (1.6)	**0.027**
Trust and hope	6.7 (2.1)	7.6 (2.1) *n* = 9	0.086	5.7 (1.7)	6.0 (1.9)	0.512
**Mean (SD) MHRS networks**	6.3 (1.6)	6.9 (1.9)	0.255	5.0 (1.9)	5.5 (2.0)	**0.019**
Social networks	7.0 (2.3)	7.3 (1.9) *n* = 9	0.608	4.5 (1.5)	4.9 (1.8)	**0.028**
Relationships	5.6 (2.4)	6.4 (2.5) *n* = 9	0.198	5.3 (1.5)	5.9 (1.6)	**0.033**

^*^p-value < 0.05 Bold values denote statistical significance at the *p* < 0.05 level

### Progress in recovery and changes in recovery goals from recruitment to 6 month follow up

Table 5 also shows that from recruitment to follow-up, both men and women made progress in their recovery in terms of increases in ratings on the MHRS Scale of Change but remained in the same recovery phase (women ‘Learning Phase’, men ‘Believing Phase’). This increase was statistically significant for men but not women.

There were statistically significant increases in scores in the MHRS dimension of ‘activities and functioning’ for women, (and a marginal improvement in the domain ‘physical health’), whereas there were statistically significant improvements in ratings for men across all four MHRS domains and multiple sub-domains (Table 5).

At 6 months follow-up, 80.0% of the service user-key professional/case manager diads completed intervention plans addressing the domains they had identified together when completing the MHRS at recruitment. ‘Activities and functioning’ remained the most commonly chosen goal (16, 80.0%) and self-image remained the least unpopular (3,15.0%) but there were shifts in choices for men and women; fewer women chose ‘networks’ than at recruitment (3, 30.0%) and more men chose ‘physical and mental health’ (12, 85.7%) (Table 4).

## Discussion

This is the first Italian study to investigate differences in the personal recovery process between men and women users of a community mental health service and whether the use of a collaborative tool such as the MHRS can facilitate an individualised approach to care planning for men and women.

Although 4 to 8% of the general population identifies as gender diverse, all service users in our study identified as either male or female. This could be due to prevailing socio-cultural norms and possible apprehension of disclosing gender diversity [57].

Men and women in our study had similarly severe mental health problems as reflected in their diagnoses and ratings of their clinical status and functioning at recruitment. Women were rated as further on in their recovery than men at recruitment and there were differences between the genders in their chosen recovery goals. Women tended to want to focus on social networks while men tended to focus on activities and working towards employment. It is plausible that these choices were influenced by gender based expectations [58]. Social norms for men and women in Italy have, traditionally, been more ‘gendered’ than some other European countries, with men expected to be the ‘bread winners’ and women expected to take care of the home and family life [59, 60]. Our participants’ choices may therefore reflect these social norms [22, 61]. Given that employment is considered in Italy one of the principal signifiers of adult life above all for men [58, 62] and income and social rights are generally linked to working status, this focus on employment by men is understandable [63]. However, it is of interest that six months later, women were no longer favouring ‘networks’ as much, perhaps because some had worked successfully towards this recovery goal, as evidenced by the fact that they had formed romantic partnerships. Similarly, there were gains for men in terms of engagement with employment related activities. Interestingly, women and men also progressed in areas which were not the main focus of their collaborative intervention plans at recruitment, demonstrating that personal recovery-based practice can extend into other aspects of the therapeutic work. Furthermore, at six months follow-up, participants showed improvement in functional autonomy and men also improved on other measures of psychopathology and functioning.

### Strengths and limitations

While this study offers some suggestions into the relationship between gender and recovery-oriented services, we recognize that further investigation is necessary to fully understand the complex interplay between gender, culture, and recovery in mental health services. First of all, this study was small and exploratory and our findings must therefore be treated with caution. Gains on the MHRS and other outcome tools may reflect natural improvement over time, unrelated to the use of the collaborative care planning tool (regression to the mean effect). A further limitation of this study is that the results cannot be generalized to other settings, especially since there were group differences regarding educational status, diagnosis, and substance abuse and since it was conducted in only one mental health service.

Furthermore, no service users were involved in the study design. In addition, multiple statistical testing reduces the statistical power of the study and our results can only give an indication of possible effects. Further studies with appropriate control groups are required to investigate whether the use of a collaborative care planning tool increases the effectiveness of the service and enhances gender sensitivity.

## Conclusions

Our findings suggest that collaborative care planning tools such as the MHRS can assist in identifying individualized recovery goals for men and women with severe mental health problems as part of their rehabilitation and recovery. This finding suggests that collaborative care planning might empower a more gender sensitive approach to providing appropriate support to people with severe mental health problems.

Further, larger controlled studies are essential to gain a deeper understanding of the relationship between gender and personal recovery. In addition, studies that include more diverse samples would also be valuable.

## Data Availability

Requests for original (fully anonymised) participant data may be made to the corresponding author.

## Abbreviations

CAN: Camberwell Assessment of Need
CMHS: Community mental health service
FPS: Personal and Social Functioning Scale
HoNOS: Health of the Nation Outcome Scale
MHRS: Mental Health Recovery Star
MPR: Monitoring of the Path of Rehabilitation
